# Retracted: Coronary Angiography Safety between Transradial and Transfemoral Access

**DOI:** 10.1155/2018/3045963

**Published:** 2018-07-19

**Authors:** Cardiology Research and Practice

Cardiology Research and Practice has retracted the article titled “Coronary Angiography Safety between Transradial and Transfemoral Access” [1]. The article was found to contain a substantial amount of material from the following published articles:

  C. M. S. Kabir, M. M. Haq, S. R. Khan, M. Z. Chowdhury, M. L. Ali, and M. R. Karim, “Safety of radial vs. femoral artery access in coronary angiography,” Bangladesh Heart Journal, vol. 30, no. 2, pp. 68–73, 2016, doi: 10.3329/bhj.v30i2.28814 (not cited).
  M. Brueck, D. Bandorski, W. Kramer, M. Wieczorek, R. Höltgen, and H. Tillmanns, “A randomized comparison of transradial versus transfemoral approach for coronary angiography and angioplasty,” JACC: Cardiovascular Interventions, vol. 2, no. 11, pp. 1047–1054, 2009, doi: 10.1016/j.jcin.2009.07.016 (not cited).

There are also concerns with the reporting of the study design. The methods state “It was prospective, randomized, single-centre study conducted in the Department of Cardiology, LPS Institute of Cardiology, GSVM Medical College, Kanpur, U.P, India, where all cases of diagnostic coronary angiography (CAG) of 1997 consecutive patients for various reasons over a 12-month period (from January 2015 till the end of December 2015) were reviewed for this analysis,” but then that “The choice between transfemoral or transradial artery access was operator's discretion with right radial approach being the default strategy.” These are mutually exclusive approaches to treatment allocation, that is, randomization versus surgeon discretion. Additionally, a trial registration number is not included.

We were unable to contact the authors.
